# Endogenous Follistatin-like 1 guarantees the immunomodulatory properties of mesenchymal stem cells during liver fibrotic therapy

**DOI:** 10.1186/s13287-022-03042-4

**Published:** 2022-08-05

**Authors:** Xiaohong Zheng, Xia Zhou, Gang Ma, Jiahao Yu, Miao Zhang, Chunmei Yang, Yinan Hu, Shuoyi Ma, Zheyi Han, Wen Ning, Boquan Jin, Xinmin Zhou, Jingbo Wang, Ying Han

**Affiliations:** 1 State Key Laboratory of Cancer Biology, Xijing Hospital of Digestive Diseases, Fourth Military Medical University, 127 Changle West Road, Xi’an, 710032 China; 2 Department of Immunology, Fourth Military Medical University, Xi’an, 710032 China; 3 State Key Laboratory of Medicinal Chemical Biology, College of Life Sciences, Nankai University, Tianjin, 300071 China

**Keywords:** FSTL1, Mesenchymal stem cells, Liver cirrhosis, Cell therapy, Immunosuppressive

## Abstract

**Background:**

Mesenchymal stem cell (MSC) therapy has been shown to be a promising option for liver fibrosis treatment. However, critical factors affecting the efficacy of MSC therapy for liver fibrosis remain unknown. Follistatin-like 1 (FSTL1), a TGF-β-induced matricellular protein, is documented as an intrinsic regulator of proliferation and differentiation in MSCs. In the present study, we characterized the potential role of FSTL1 in MSC-based anti-fibrotic therapy and further elucidated the mechanisms underlying its action.

**Methods:**

Human umbilical cord-derived MSCs were characterized by flow cytometry. FSTL1^low^ MSCs were achieved by FSTL1 siRNA. Migration capacity was evaluated by wound-healing and transwell assay. A murine liver fibrotic model was created by carbon tetrachloride (CCl_4_) injection, while control MSCs or FSTL1^low^ MSC were transplanted via intravenous injection 12 weeks post CCl_4_ injection. Histopathology, liver function, fibrosis degree, and inflammation were analysed thereafter. Inflammatory cell infiltration was evaluated by flow cytometry after hepatic nonparenchymal cell isolation. An MSC-macrophage co-culture system was constructed to further confirm the role of FSTL1 in the immunosuppressive capacity of MSCs. RNA sequencing was used to screen target genes of FSTL1.

**Results:**

FSTL1^low^ MSCs had comparable gene expression for surface markers to wildtype but limited differentiation and migration capacity. FSTL1^low^ MSCs failed to alleviate CCl_4_-induced hepatic fibrosis in a mouse model. Our data indicated that FSTL1 is essential for the immunosuppressive action of MSCs on inflammatory macrophages during liver fibrotic therapy. FSTL1 silencing attenuated this capacity by inhibiting the downstream JAK/STAT1/IDO pathway.

**Conclusions:**

Our data suggest that FSTL1 facilitates the immunosuppression of MSCs on macrophages and that guarantee the anti-fibrotic effect of MSCs in liver fibrosis.

**Supplementary Information:**

The online version contains supplementary material available at 10.1186/s13287-022-03042-4.

## Background

Liver diseases are a global health issue that are associated with high mortality and morbidity worldwide [1, 2]. Liver fibrosis, which occurs as a general response to chronic liver injury, may progress to cirrhosis when not well controlled [3, 4]. Although liver transplantation is the most effective treatment for patients with end-stage cirrhosis, the high costs and shortage of donors limit the clinical application [5]. Recently, following the publication of studies showing the significant efficacy in animal models and preliminary clinical trials, mesenchymal stem cell (MSC) therapy has been shown to be a promising option for liver fibrosis. However, factors regulating the efficacy of MSC therapy for liver fibrosis are not completely understood [6, 7].

MSCs were first isolated and identified by Friedenstein et al. [8] from the bone marrow and have subsequently been isolated from various tissues [9]. The multi-lineage development, immunomodulatory, and trophic properties of MSCs make these cells ideal candidates for clinical applications [10]. During chronic injury, proinflammatory macrophages trigger hepatic stellate cells (HSCs) activation and differentiation into collagen-producing myofibroblasts [11]. Persistent hepatic inflammation activates HSCs to produce extracellular matrix (ECM) and precedes the progression of liver fibrosis [12]. Switching of macrophages from a proinflammatory to an anti-inflammatory phenotype is believed to underlie the therapeutic effects of MSCs in treating liver fibrosis [6]. This function mainly relies on the factors and molecules secreted by MSCs, including Indoleamine 2,3-dioxygenase (IDO), NO, TSG6 and PGE2, which are linked to the immunosuppressive function of MSCs. However, factors which regulate the immunosuppression of MSCs are largely unknown.

Follistatin-like protein 1 (FSTL1), a TGF-β–induced extracellular glycoprotein belonging to the SPARC family of matricellular proteins [13, 14], functions as an important modulator of cell–matrix interactions by integrating signalling networks that regulate essential cell functions. FSTL1 is involved in development, tissue remodelling/repair, and inflammatory processes [15, 16]. Previous studies have shown that FSTL1 could regulate MSC proliferation and chondrogenic differentiation [16–18]. Shen et al. [19] and Zhang et al. [20] reported that FSTL1 is an intrinsic cardiokine promoting survival and proliferation of MSC, and facilitates their therapeutic efficacy in a myocardial ischemia model. Kim et al. [21] found that FSTL1, downregulated in the MSC secretome from patients with cerebellar ataxia, could serve as an anti-inflammatory cytokine with suppressive effects on proinflammatory microglial activation. Paradoxically, it is worth noting that FSTL1 has been identified as a novel inflammatory protein that enhances the ability of monocytes/macrophages to respond to inflammatory signals [16, 17, 22]. FSTL1 acts as a pro-fibrotic factor in organs, including the liver, in the case of liver fibrosis [22–26]. Based on these findings, we focused on the role of intrinsic FSTL1 in MSCs for the treatment for liver fibrosis.

In the present study, we knocked down FSTL1 in MSCs and observed the abolishment of MSC-mediated anti-fibrotic therapy in carbon tetrachloride (CCl_4_)-induced liver fibrosis. Our data suggest that FSTL1 facilitates the immunosuppression of MSCs on macrophages and guarantees the anti-fibrotic effect of MSCs in liver fibrosis.

## Materials and methods

### Methods

#### Animals and cells

Male C57BL/6 J mice (6–8 weeks) were purchased and housed in the animal centre of The Fourth Military Medical University, in a pathogen-free airflow cabinet and food and water were provided ad libitum. All animal study protocols were approved by the Animal Welfare and Ethics Committee of the Fourth Military Medical University (FMMU) and performed in accordance with the ‘Guidelines for the Care and Use of Laboratory Animals.’ The murine liver fibrosis model was established by intraperitoneal injection of 7 ml/kg [20% (v/v)] CCl_4_ twice a week for 12 weeks. Subsequently, fibrotic mice were randomly divided into groups (*n* = 6) that received phosphate buffered saline (PBS), 1 × 10^6^ control MSCs, and FSTL1^low^ MSCs (MSCs transfected with FSTL1 siRNA) by tail vein injection. Mice were continually injected with CCl_4_ during the observation period and were sacrificed at the indicated time for sectioning and analysis.

hMSCs were obtained from the National Engineering Research Centre (Tianjin AmCellGene Engineering Co., Ltd, China) and were passaged in the MSC expansion medium (CCM004, R&D Systems, MN) with 5% CO_2_ and saturated humidity at 37 °C. hMSCs in the 4th to 10th passages were used in all experiments and for all in vitro evaluation of MSCs. The characterization of hMSCs was performed using flow cytometry. Antibodies (anti-CD29, CD90, CD105, CD73, CD44, CD45, CD34, and HLA-DR) conjugated with PE were purchased from BioLegend (San Diego, CA). Analyses were performed using FACScan (BD Bioscience, CA). hMSCs were cells positive for CD105, CD44, CD29, and CD90, but negative for CD34 and CD45 (Additional file 1: Fig. S1). Osteoblast, adipocyte, and chondrogenic differentiation media were purchased from Cyagen Biosciences (HUXUC-90021, HUXUC-90031, HUXUC-9004, Guangzhou, China) and applied according to the manufacturer’s instructions. Intracellular lipid or calcium deposits were stained using Oil Red O or Alizarin Red S. The presence of proteoglycans, which is indicative of chondrogenic differentiation, was verified by toluidine blue after 21 d of pellet induction in a 15 mL tube. Differentiation assays showed that MSCs could differentiate into adipocytes, osteocytes, and chondrocytes under conditioned medium (Additional file 1: Fig. S1).

### Transfection of MSCs with FSTL1-siRNA

The siRNAs against FSTL1 were purchased from Santa Cruz Biotechnology, Inc (USA). One day before transfection, MSCs were seeded in a 6-well plate at a density of 1 × 10^5^ cells/mL. For treatment, 2 μL siRNA was diluted in 120 μL 1 × ribo*FECT™* CP Buffer. Then, 12 μL ribo*FECT™* CP Reagent was added and incubated for 15 min. Subsequently, the mixture was diluted in DMEM containing 10% (v/v) serum and added into the wells containing MSCs. After 24–48 h, the cells were harvested for mRNA and protein analysis.

### RNA isolation and real-time PCR analysis

Total RNA was extracted using the RNAeasy Plus kit (TaKaRa Biotechnology Co., Ltd., Dalian, China), and reverse transcription was performed using the PrimeScript™ RT Master Mix (RR036A, Takara, Tokyo). Amplification was conducted using TB Green Premix Ex Taq II (DRR820A, Takara, Tokyo) and a CFX96 Touch™ real-time PCR System (Bio-Rad, CA).

### Western blot analysis

Proteins were extracted from cells or tissue samples using RIPA lysis buffer (Beyotime biotechnology, China), which was supplemented with proteinase inhibitors and phosphatase inhibitors (Roche, Basel). The Bradford method was applied to quantify the protein samples. Subsequently, 30 μg of protein was loaded onto SDS-PAGE gels before transferring to nitrocellulose membranes (Bio-Rad Biotechnology, America). The membranes were blocked in TBST buffer containing 2.5% skim milk for 30 min. Subsequently, the membranes were incubated with primary antibody at 4 ℃ overnight. The next day, the membranes were washed and incubated with peroxidase-conjugated secondary antibodies at room temperature for 1 h. Blots were imaged using an enhanced chemiluminescence kit.

### Wound healing assay

Cells were seeded onto 6-well plates and transfected with siFSTL1 24 h before treatment. The monolayers were scratched with a 200 mL pipette tip and washed with serum-free media to remove the detached cells. The cells were maintained in serum-free media for 12 h. The wound areas were then imaged.

### Transwell migration assay

The migration ability of cells was determined using a transwell membrane system comprising an 8 μm pore polycarbonate membrane in a 24-well culture plate. In brief, MSCs were cultured in the upper chamber of a transwell membrane system without serum (1 × 10^5^ cells per well). The lower chamber was filled with 600 μL medium containing 20% serum. Following a 12 h incubation, the cells in the upper chamber were fixed with 4% paraformaldehyde for 30 min, and the non-migrating cells inside the chamber were detached from the membrane. Subsequently, crystal violet was used to stain the migrated cells in the chamber. Finally, the images of stained cells were acquired and counted in five random fields.

### Biochemical analysis and histological staining

The serum of mice was obtained at each time point. The levels of alanine aminotransferase (ALT) were analysed using an automatic biochemistry analyser in Xijing Hospital. The liver tissue samples were prepared as paraffin-embedded sections by YiKE Biotechnology, China. Subsequently, the sections were stained with haematoxylin and eosin (HE) for routine histological examination and Sirius Red for fibrosis evaluation. Further quantification and analysis of the collagen fibres were performed using Image-Pro Plus software (v6.0, Media Cybernetics Inc.).

### Immunohistochemistry staining

The liver tissue samples were prepared as paraffin-embedded sections by YiKE Biotechnology, China. Subsequently, the sections were dewaxed and sequentially rehydrated by in xylol and alcohol in sequence. Antigen retrieval was achieved by microwave using sodium citrate solution with pH 6.0. Subsequently, 1% H2O2 was used to block endogenous peroxidase activity. The slides were further blocked in goat serum for 30 min. Anti-iNOS and anti-CD206 antibodies were incubated with the slides at 4 °C overnight. The next day, after washing with PBS, the secondary antibodies were added and diaminobenzidine (DAB) was applied to visualise the images. Further quantification and analysis of the positive areas were performed using Image-Pro Plus software (v6.0, Media Cybernetics Inc.).

### Flow cytometry analysis of liver leukocyte subsets

Hepatic mononuclear cells were isolated from the liver by using collagenase IV (Sigma) and 40% Percoll (GE Healthcare). Single-cell suspensions were first incubated with anti-mouse FcR blocking reagent (BioLegend) and then labelled with mixed fluorochrome-conjugated antibodies, (APC/Cyanine 7 anti-mouse CD45.2, PE anti-mouse F4/80, Brilliant Violet 421 anti-mouse CD206 (MMR), APC anti-mouse CD86, FITC anti-mouse/human CD11b, PerCP/Cyanine5.5 anti-mouse CD11c, and APC anti-mouse I-A/I-E). Flow cytometric data were acquired on a FACSVerse flow cytometer (BD Bioscience) and analysed using FlowJo software (TreeStar, Ashland, OR, US).

### RNA sequencing (RNA-seq) analysis

MSCs were transfected with FSTL1 siRNA for 24 h, and then treated with or without FSTL1 (100 ng/ml) for 24 h. Cells were washed with PBS for 3 times. Total RNA was isolated from control MSCs, MSCs transfected with siRNA treated with or without FSTL1 using Trizol (Invitrogen, USA) according to the manual instruction. RNA sequencing libraries were generated with an insert size ranging from 370 to 420 bp, and sequenced using the Illumina NovaSeq 6000 platform (Novogene-Tianjin, China). The image data measured by the high-throughput sequencer are converted into sequence data (reads) by CASAVA base recognition. Raw data (raw reads) of fastq format were firstly processed through in-house perl scripts. In this step, clean data (clean reads) were obtained by removing reads containing adapter, reads containing N base and low quality reads from raw data. At the same time, Q20, Q30 and GC content of the clean data were calculated. All the downstream analyses were based on the clean data. Each sample produced 15.0 G data on average. The clean reads were mapped to the reference genome using HISAT2 (v2.0.5) software. Data processing and analysis were performed using the R programming language. Quantification of gene expression level featureCounts (v1.5.0-p3) was used to count the reads numbers mapped to each gene. And then FPKM of each gene was calculated based on the length of the gene and reads count mapped to this gene. FPKM, expected number of Fragments Per Kilobase of transcript sequence per Millions base pairs sequenced. (For DESeq2 with biological replicates) Differential expression analysis of two conditions/groups (two biological replicates per condition) was performed using the DESeq2 R package (1.20.0). DESeq2 provides statistical routines for determining differential expression in digital gene expression data using a model based on the negative binomial distribution. The resulting P-values were adjusted using the Benjamini and Hochberg’s approach for controlling the false discovery rate. padj <  = 0.05 and |log2(foldchange)|> = 1 were set as the threshold for significantly differential expression. Differentially expressed genes (DEGs) were included for further functional analysis based on GO and KEGG databases. The details of all the identified genes were listed in Additional file 2: Table 2.

### Statistical analysis

Data were expressed as the mean values ± SD. One-way analysis of variance and a t-test were performed to identify the significant differences. A *P* value of < 0.05 was considered significant. Statistical analysis was performed using GraphPad Prism 7.0 (GraphPad Software, CA).

## Results

### Generation of FSTL1^low^ MSCs

**Fig. 1:**
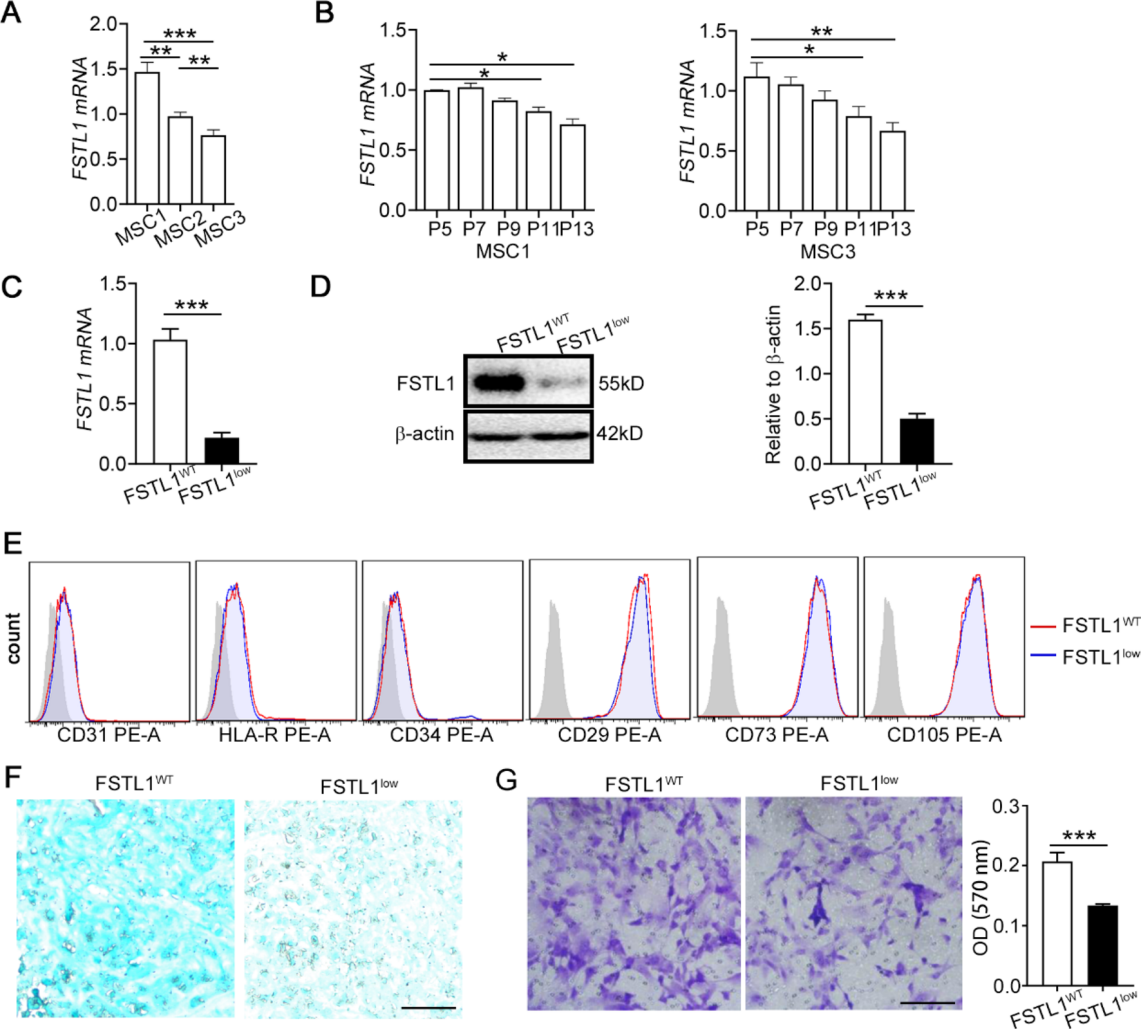
Generation of human umbilical cord-derived FSTL1 ^low^ MSCs. *A-B:* FSTL1 level was evaluated by qPCR in human umbilical cord-derived MSCs. FSTL1 expression in MSCs in passage 3 from different origin (**A**). FSTL1 expression in MSCs after grow for eight passages (**B**). *C-E:* MSCs were transfected with siFSTL1 for 12 h prior to RNA analysis, or 24 h for protein or flow cytometry (FCM) analysis. FSTL1 mRNA levels were determined with qRT-PCR (**C**, *n* ≥ 5, **, *P* < 0.01). Western blot analysis of FSTL1 in cell extracts (**D**). Relative density of FSTL1-normalised β-actin is represented. FCM analysis of surface markers after FSTL1 silencing (**E**). *F-G*: MSCs were transfected with siFSTL1 for 24 h, followed by differentiation or migration capacity evaluation. The ability of MSCs to differentiate into the chondrogenic was confirmed by Alcian blue staining (**F**). The migration ability of MSCs was evaluated by transwell assays (**G**). ***, *P* < 0.001; **, *P* < 0.01; *, *P* < 0.05. Bars, 100 µm. All experiments were performed three times. Error bars indicate the mean ± SEM

As FSTL1 expression is robust in most types of mesenchymal cells, we evaluated FSTL1 mRNA in subsequent passages in human umbilical cord-derived MSCs (hMSCs). We found that Fstl1 expression was different in MSCs from different origin (Fig. 1A). And it was generally downregulated in MSCs after growth for over ten passages (Fig. 1B, Additional file 1: Fig.S2A). To study the function of FSTL1 on the therapeutic effect of MSCs, FSTL1^low^ MSCs were generated using RNA interference in MSCs to knock down endogenous FSTL1 (Fig. 1C, D). FSTL1^low^ MSCs showed no obvious change in expression of CD surface-markers compared with controls (Fig. 1E), but they significantly inhibited osteogenesis and chondrogenesis as previously reported [27] (Fig. 1F, Additional file 1: Fig. S2B). Further, we observed that FSTL1^low^ MSCs had attenuated migration capacity in wound healing (Additional file 1: Fig. S2C) and transwell (Fig. 1G) assays. These results indicate that silencing of FSTL1 does not affect MSC identity, but inhibits their differentiation and migration capacity.

### FSTL1^low^ MSCs had impaired treatment efficacy in liver fibrosis

**Fig. 2:**
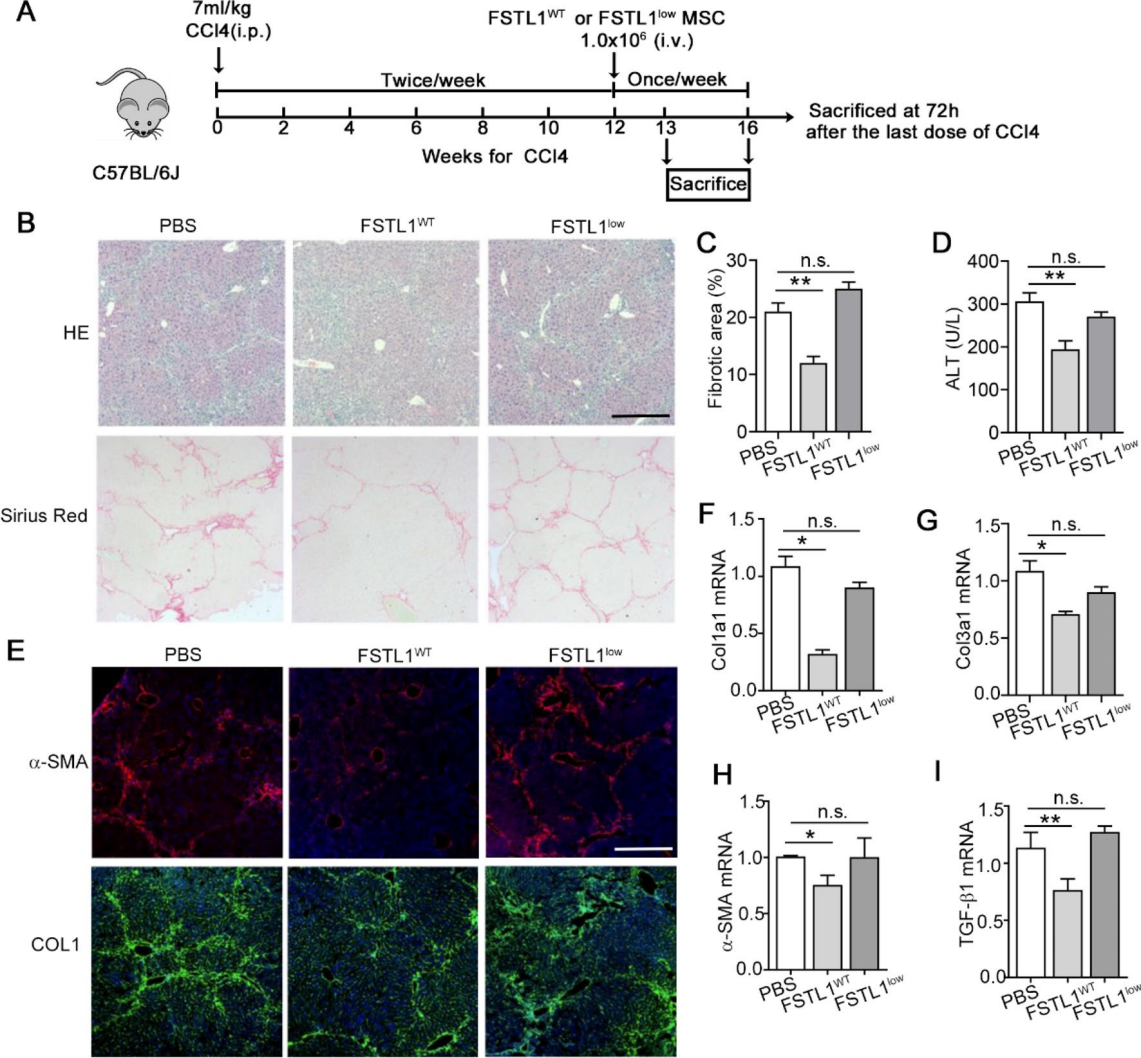
FSTL1^low^ MSCs showed impaired treatment capacity in liver fibrosis. **A** Schematic illustration of the establishment of an in vivo hepatic fibrosis model and the MSC-based treatment strategy. MSC transplantation was performed at 12 weeks post CCl_4_ injury, livers were harvested at 4 weeks post transplantation for subsequent analysis. **B** Histological analysis and Sirius red staining of collagen in liver sections. Representative images of the staining are shown (*n* = 4). **C** Liver fibrosis score analysis of Sirius red-stained liver sections. The fibrotic area is presented as a percentage. **D** Serum ALT changes after MSC treatment. **E**–**G**: Immunofluorescent staining (**E**) and qRT-PCR analyses (**F**–**I**) of fibrotic markers. Representative images of the staining are shown (*n* = 4). **, *P* < 0.01; *, *P* < 0.05. Bars, 100 µm. All experiments were performed three times. Error bars indicate the mean ± SEM

**Fig. 3:**
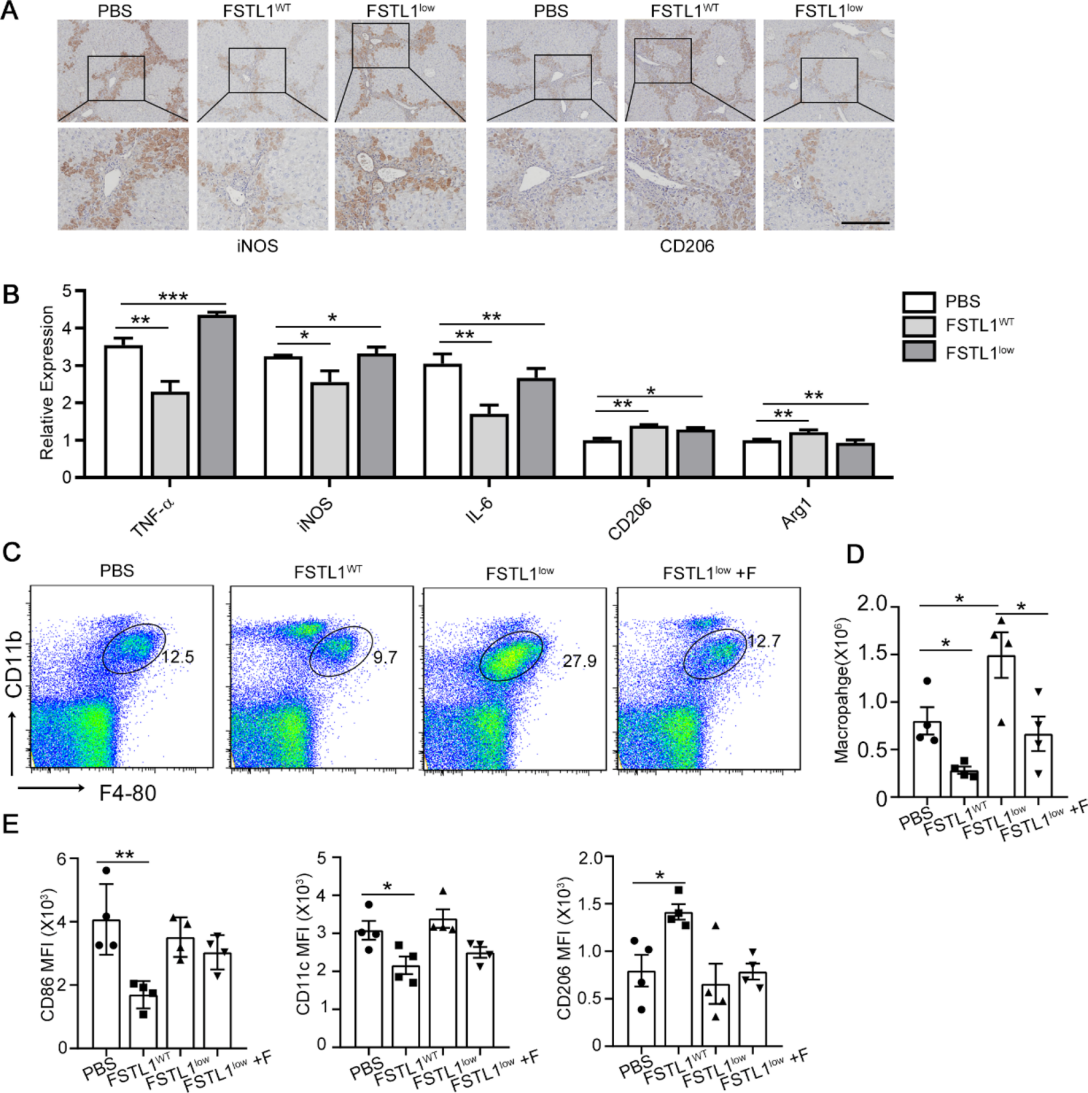
FSTL1^low^ MSCs lose their immunosuppressive capacity on inflammatory macrophages in fibrotic mouse model. **A** and **B**: Infiltrating inflammatory macrophages and cytokines in liver tissue were evaluated 1 weeks after MSCs transplantation. Pro-inflammatory macrophages (iNOS +) and anti-inflammatory macrophages (CD206 +) were analysed using IHC staining (**A**). Cytokines in liver tissues related to inflammatory macrophages were evaluated by qRT-PCR analyses (**B**). **C**–**E**: Non-parenchymal cells in livers were isolated and analysed at 1 week post MSC transplantation for subsequent analyses. Total number of macrophages (F4/80 + CD11b +) in the fibrotic liver (**C** and **D**, *n* = 4). (E) Mean fluorescence intensity (MFI) of inflammatory surface markers on macrophages (CD11c, CD206, CD86) after MSC treatment (*n* = 4). **, *P* < 0.01; *, *P* < 0.05. Bars, 100 µm. All experiments were performed in triplicate. Error bars indicate the mean ± SEM

To further assess the role of intrinsic FSTL1 in MSCs for their anti-fibrotic efficacy, we transplanted FSTL1^low^ MSCs via tail vein into mice with CCl_4_-induced liver fibrosis (Fig. 2A). Histological analyses of liver tissues showed that mice transplanted with control MSCs exhibited remarkably reduced fibrosis, while those transplanted with FSTL1^low^ MSCs did not show any pronounced amelioration at 4 weeks post transplantation (Fig. 2B). The area of Sirius Red staining (Fig. 2C) and serum parameters (alanine aminotransferase, ALT) that indicated hepatic function restoration (Fig. 2D) was barely altered at 4 weeks post FSTL1^low^ MSC transplantation. Immunofluorescence of α-SMA and Col1, both markers of activated myofibroblasts, revealed a significant decrease in the MSC-transplanted group, but no obvious changes in the FSTL1^low^ MSC-transplanted group (Fig. 3E). Consistent with these results, FSTL1^low^ MSC treatment did not inhibit the transcript-level expression of fibrotic genes (Col1al, Col3a1,α-SMA,, TGF-β1,) in the liver tissue, while the expression of genes in the control group was markedly decreased (Fig. 3F–I). These findings suggested the indispensable role of FSTL1 in MSC antifibrotic therapy in the context of liver fibrosis.

### FSTL1^low^ MSCs lose their immunosuppressive capacity on inflammatory macrophages in liver fibrosis mouse model

With a crucial role in the pathogenesis of chronic liver injury, hepatic macrophages have been proposed as potential therapeutic targets in fibrosis [11]. It has further been found that liver macrophage depletion ameliorates the effect of MSC transplantation, indicating the importance of macrophages for MSC therapy [28]. As previously reported [29], MSC transplantation significantly decreased the amount of iNOS-positive pro-inflammatory macrophages and increased the number of CD206-positive anti-inflammatory macrophages in liver Sects. 1 week post MSCs transplantation (Fig. 3A). However, the same improvement was not observed in the FSTL1^low^ MSC-transplanted group. Furthermore, consistent with these findings, in the FSTL1^low^ MSC-transplanted group, expression of genes related to the pro-inflammatory function of macrophages (TNF-α, iNOS and IL-6) showed no obvious downregulation and there was no increase in the anti-inflammatory function of macrophages (indicated by CD206 and Arg1 expression) (Fig. 3B). Flow cytometry also showed that FSTL1^low^ MSCs did not sufficiently inhibit macrophage infiltration (Fig. 3C, D). Further, FSTL1^low^ MSCs exerted no obvious suppression of pro-inflammatory markers (CD86, CD11c) or elevation of anti-inflammatory markers (CD206), as was observed in the MSC-transplanted group (Fig. 3E). FSTL1^low^ MSCs partially restore their immunosuppressive capacity after FSTL1 addition in culture system. These data suggest that FSTL1 is essential for MSC immunosuppression on pro-inflammatory macrophages during liver fibrosis therapy.

### FSTL1^low^ MSCs lose their immunosuppressive function towards macrophages in vitro

**Fig. 4:**
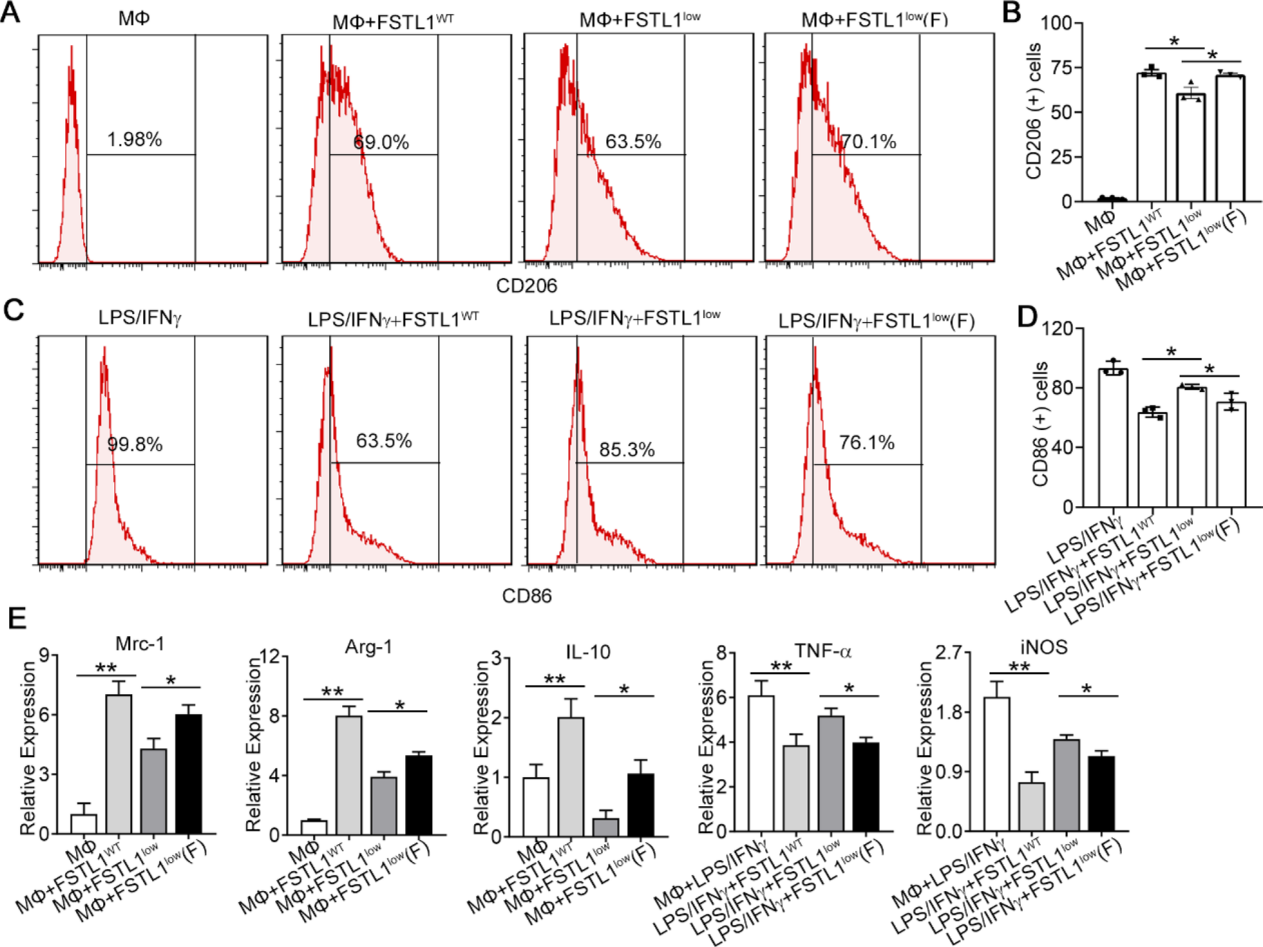
FSTL1^low^ MSCs lose their immunosuppressive function on macrophages in vitro. *A-B*: MSC transfected with siFSTL1treated with or without FSTL1 (100 ng/ml) were co-cultured with Raw264.7 cells. The proportions of CD206-positive macrophages were determined after 24 h co-culture with MSCs (**A**). The mean percentages of CD206-positive cells in total macrophages were summarised (**B**). **C**, **D**: MSCs transfected with siFSTL1 treated with or without FSTL1 (100 ng/ml) were co-cultured with Raw264.7 cells after 24 h IFN-γand LPS treatment. The proportions of CD86-positive cells were subsequently determined (**C**). Mean percentages of CD86-positive cells in total macrophages were summarised after 24 h co-culture with MSCs (**D**). **E** Expression of differential markers in macrophages evaluated by qPCR under different conditions after 12 h co-culture with MSCs. **, *P* < 0.01; *, *P* < 0.05. All experiments were performed in triplicate. Error bars indicate the mean ± SEM

To further explore the effect of intrinsic FSTL1 of MSCs in their immunoregulatory function, we performed an in vitro MSC-macrophage co-culture assay. Raw264.7, a mouse monocyte/macrophage cell line, co-cultured with hMSCs. Flow cytometry of macrophage populations indicated an increase in scavenger receptor CD206-expressing cells in the MSC-macrophage co-culture system. However, macrophages from FSTL1^low^-MSC co-cultures showed no significant induction of the population of CD206-positive cells (Fig. 4A, B). Then, Raw264.7 was treated with pro-inflammatory macrophage (M1) stimuli (LPS and IFN-γ). Flow cytometry of macrophage populations indicated a reduction in co-stimulatory molecule CD86-expressing cells in this MSC-macrophage co-culture system (Fig. 4C, D). In contrast, macrophages from FSTL1^low^-MSC co-cultures showed no significant suppression of the population of CD86-positive cells. Moreover, FSTL1^low^ MSCs were inefficient with respect to inducing the expression of anti-inflammatory cytokines, Mrc-1, Arg-1 and interleukin (IL)-10, and incapable of attenuating the expression of prototypic pro-inflammatory cytokines, TNF-α and iNOS (Fig. 4E). FSTL1^low^ MSCs partially restore their immunosuppressive capacity after FSTL1 addition in culture system in both situations. These results further demonstrated that FSTL1 silencing attenuates the immunosuppressive capacity of MSCs against inflammatory macrophages.

### FSTL1 downregulation induces transcriptional reprogramming in MSCs

**Fig. 5:**
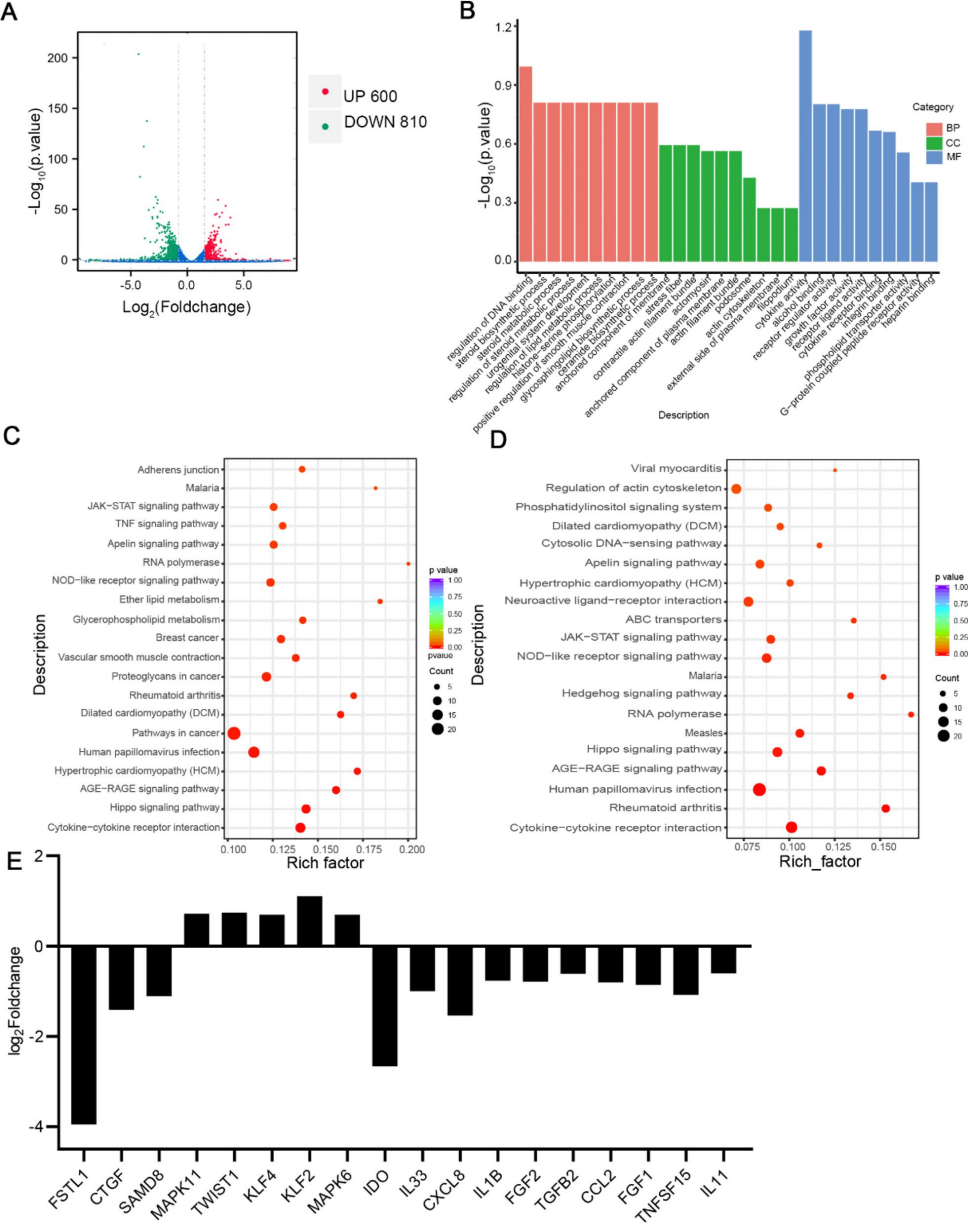
FSTL1 downregulation induces transcriptional reprogramming in MSCs. **A** Volcano plot showing DEGs in FSTL1^low^ MSCs compared to FSTL1^WT^ MSCs. The blue and red dots indicate downregulated and upregulated genes, respectively. Fold change > 1.5 and P value < 0.05) **B** Gene ontology (GO) analysis of the DEGs categorised as ‘Cellular component,’ ‘Molecular function,’ and ‘Biological process.’ **C** KEGG pathway enrichment analysis of the DEGs. The Y-axis represents KEGG terms, and the *X*-axis represents the rich factor. The colour of the bubble represents the enrichment significance, and the size of the bubble represents the number of DEGs. **D** The top downregulated enriched terms in KEGG pathway are presented as a bubble chart. **E** Representative differentially expressed genes (DEGs) between FSTL1^WT^ MSC and FSTL1^low^ MSC

To elucidate whether the expression of FSTL1 influences the gene expression profiles of MSCs, we performed RNA-seq analysis on FSTL1^WT^ and FSTL1^low^ MSCs. In total, we identified 1410 differentially expressed genes (DEGs) after FSTL1 silencing (600 upregulated genes and 810 downregulated genes) (Fig. 5A). With respect to functional analysis based on the gene ontology (GO) database, the DEGs were annotated into multiple subdivisions within the three GO categories, ‘Biological process,’ ‘Cellular component,’ and ‘Molecular function,’ implying distinct transcriptional changes induced by FSTL1 silencing (Fig. 5B).

Enrichment analysis revealed that FSTL1 silencing resulted in increased expression of genes associated with cell cycle (histone-serine phosphorylation, DNA binding and phospholipase activity) (Additional file 1: Fig. S3), and a decreased expression of genes associated with signalling transduction (cytokine receptor binding, cytokine activity, receptor complex, receptor regulator activity) (Additional file 1: Fig. S4). KEGG pathway analysis further revealed an obvious enrichment of DEGs associated with multiple inflammatory-related pathways, including “JAK/STAT signalling pathway”, “TNF signalling pathway,” and “MAPK signalling pathway”, “NOD-like receptor signalling pathway”, indicating that FSTL1 functions as a modulator of MSC behaviours and functions by integrating signalling networks (Fig. 5C). And genes associated with JAK/STAT signalling pathway were downregulated after FSTL1 silencing (Fig. 5D), which had been reported to empower the immunoregulation capacity of MSCs after cytokines stimuli. Representative DEGs that changed after FSTL1 downregulation are shown in Fig. 5E. These data indicate that silencing of FSTL1 induces the transcriptional reprogramming of MSCs.

### FSTL1 guarantees MSC immunosuppressive function by regulating IDO expression

**Fig. 6:**
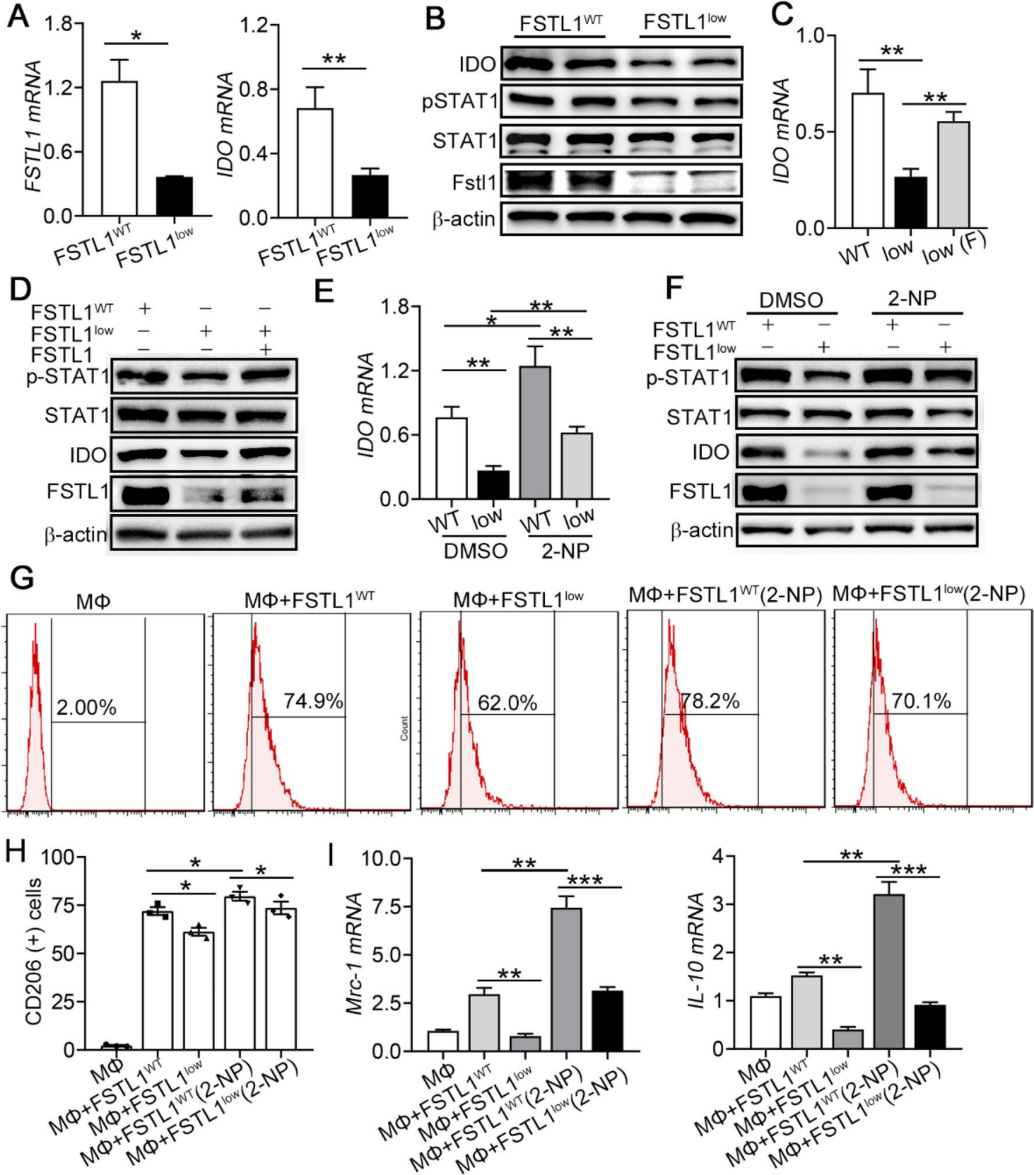
FSTL1 guarantees immunosuppressive function of MSCs on macrophages by regulating JAK/STAT1/IDO pathway. MSCs were transfected with siFSTL1. RNA expression of FSTL1 and IDO was determined by qRT-PCR at 12 h post transfection (**A**). Protein level of FSTL1, IDO, and pSTAT1 was determined by western blotting at 24 h post transfection (**B**). **C**–**D**: MSC transfected with siFSTL1treated with or without FSTL1 (100 ng/ml). IDO mRNA was determined by qRT-PCR at 12 h post FSTL1 treatment (**C**). Protein level of FSTL1, IDO, and pSTAT1 was determined by western blotting at 24 h post FSTL1 treatment (**D**). E–F: MSC transfected with siFSTL1treated with or without 2-NP (15 ug/ml). IDO mRNA was determined by qRT-PCR at 12 h post 2-NP treatment (**E**). Protein level of FSTL1, IDO, and pSTAT1 was determined by western blotting at 24 h post 2-NP treatment (**F**). G-I: MSC transfected with siFSTL1treated with or without 2-NP treatment were co-cultured with Raw264.7 cells. The proportions of CD206-positive macrophages were determined after 24 h co-culture with MSCs (**G**). The mean percentages of CD206-positive cells in total macrophages were summarised (**H**). Expression of differential markers in macrophages evaluated by qPCR under different conditions after 12 h co-culture with MSCs (**I**). ***, *P* < 0.001; **, *P* < 0.01; *, *P* < 0.05. All experiments were performed in triplicate. Error bars indicate the mean ± SEM

IDO, the rate-limiting enzyme in the kynurenine pathway of tryptophan degradation, plays an important role in inflammation. It has been reported that IDO can not only affect the infiltration of immune cells in many pathological disease conditions, including liver fibrosis [30, 31], but also further inhibits T cell proliferation and promotes anti-inflammatory macrophage differentiation during MSC therapy [29, 32]. Based on the results of our RNA-seq analysis, we hypothesized that IDO was one of the target genes of FSTL1. Using qPCR and Western blot, we further confirmed that silencing of FSTL1 downregulated IDO at both the mRNA (Fig. 6A) and protein levels (Fig. 6B). We found that JAK/STAT1 signalling was downregulated following FSTL1 silencing. Most importantly, we found that FSTL1 promotes MSC immunosuppressive functions by activating the downstream JAK/STAT1/IDO pathway. FSTL1^low^ MSCs partially restore IDO expression and STAT1 phosphorylation after FSTL1 addition in culture system (Fig. 6C, D). 2-(1,8-naphthyridin-2-yl)-Phenol (2-NP), a STAT1 transcriptional activator, could rescue JAK/STAT1/IDO axis in FSTL1^low^ MSCs (Fig. 6E, F). Besides, we also found that 2-NP could significantly enhance immunosuppression of FSTL1^low^ MSC after co-cultured with macrophage (Fig. 6G–I). These data indicate FSTL1 promotes MSC immunosuppressive functions by activating the downstream JAK/STAT1/IDO pathway.

## Discussion

Liver disease is associated with high mortality and morbidity, with an increasing incidence worldwide. Recently, MSC transplantation is generally believed to be a promising therapeutic strategy for patients with end-stage liver disease [33]. However, it is still not recognized as a reproducible, predictable, and standardized therapeutic approach in the clinical setting [34]. Thus, factors affecting the efficacy of MSC therapy for liver fibrosis need to be fully characterized. These results provide evidence at the cellular, molecular, and animal levels to support a regulatory role of FSTL1 with respect to the immunosuppressive effect of MSCs on inflammatory macrophages, which affects the therapeutic outcome.

MSCs have a self-renewal ability and multi-directional differentiation potential. FSTL1, a multifunctional secreted protein mainly expressed in interstitial cells, has a role in maintaining MSC stemness [18]. It has been reported to participate in cell survival, proliferation, differentiation, migration, and organ development. Our work here was consistent with previous findings that FSTL1 could regulate the chondrogenic differentiation of MSCs [18]. It has been shown that Follistatin, another SPARC family member, promotes MSC migration in vitro [35]. We also found that FSTL1 could facilitate MSC migration capacity, which potentially enhances the rate of MSC homing to injured sites.

Our work further illustrates the role of endogenous FSTL1 in MSC therapy. During MSC therapy for myocardial infarction, Han et al. showed that overexpression of FSTL1 in MSCs can significantly reduce inflammation and enhance neovascularization [19]. However, as FSTL1 can be used as a pro-survival cytokine to inhibit the death of cardiomyocytes [27], it is hard to distinguish whether the therapeutic improvement in FSTL1-overexpressed MSCs depends on more activated MSCs or the molecular function of FSTL1 alone. Here, we tried to determine the role of FSTL1 in MSC therapy and investigate how it affects the treatment outcome in liver fibrosis. The data indicated that endogenous FSTL1 expression in MSCs is indispensable for MSC treatment outcomes in liver fibrosis without a tendency to promote fibrogenesis.

It was widely accepted that the anti-fibrotic effect in liver fibrosis mainly depended on the immunomodulatory capacity of MSCs, especially when they were administered via intravenous infusion [36]. MSCs have been reported to induce a shift in the polarization of pro-inflammatory macrophages (M1) towards alternative macrophages (M2) both in vivo and in vitro. This polarization is driven by the ability of MSCs to secrete soluble factors, such as IL-10, IL-1Ra, and PGE2 by enhancing the number of M2 macrophages [6]. IDO, an IFN-γ-inducible intracellular enzyme, catalyses the first step in tryptophan degradation along the kynurenine pathway, which was reported to regulate macrophage recruitment and polarization both in humans [29] and mice [31]. We identified IDO as the most significantly varied gene in FSTL1 silencing MSCs using RNA-seq, a result that we further confirmed at the transcriptional and translational levels.

As previously reported, FSTL1 regulates several other pathways controlling chondrocyte proliferation and differentiation, including those regulated by BMP4, IGF, and Wnt signalling [18]. FSTL1 enhances the activity of various intermediaries of the TGF-β pathway, which include the canonical Smad3, as well as alternative p38 MAPK and Akt [18]. Our data show that FSTL1 silencing downregulates JAK/STAT1 signalling, which directly regulates IDO transcriptional activity. Moreover, although FSTL1 silencing suppresses the activation of several signalling pathways, we found that most upregulated genes enriched in RNA-seq were those related to DNA replication, translation, and transcription. Genes related to non-coding RNA were also significantly upregulated after FSTL1 downregulation, the mechanism of which needs further work to illustrate. Above all, FSTL1 plays a critical role in the extensive crosstalk between the numerous signalling pathways that are activated in MSCs in response to stimuli. Further research to determine how FSTL1 is integrated into the signal-transduction network maintaining MSC stemness and immunoregulatory function is important to elucidate the mechanisms underlying MSC therapy.

## Conclusion

In summary, we have described that FSTL1 silencing attenuates the immunosuppressive capacity of MSCs on inflammatory macrophages by inhibiting downstream JAK/STAT1/IDO. Our data suggest that FSTL1 facilitates the immunosuppression of MSCs on macrophages and guarantee the anti-fibrotic effect of MSCs in liver fibrosis.

## Supplementary Information

**Additional file 1**. Supplemental material 1.

**Additional file 2**. Supplemental material 2.

## Data Availability

The datasets supporting the results of this article are included within the article and its additional files.

## Abbreviations

MSC: Mesenchymal stem cell
FSTL1: Follistatin-like 1
TGF-β: Ttransforming growth factor β
CCl4: Carbon tetrachloride
JAK: Janus kinase
STAT: Signal transducer and activator of transcription
IDO: Indoleamine 2,3-dioxygenase
HSC: Hepatic stellate cells
ECM: Extracellular matrix
RNA: Ribonucleic Acid
DNA: Deoxyribonucleic acid
a-SMA: Alpha-smooth muscle actin
Col1al: Collagen I
Col3a1: Collagen III
iNOS: Inducible nitric oxide synthase
TNF-α: Tumor Necrosis Factor Alpha
LPS: Lipopolysaccharides
IFN-γ: Interferon (IFN)-γ
DEGs: Differentially expressed genes
GO: Gene ontology
MAPK: MAP kinase
KEGG: Kyoto encyclopedia of genes and genomes
PGE2: Prostaglandin E2
IGF: Insulin like growth factor
2-NP: 2-(1,8-Naphthyridin-2-yl)-phenol
