# Supplementary material for: Endogenous Follistatin-like 1 guarantees the immunomodulatory properties of mesenchymal stem cells during liver fibrotic therapy
Source: Stem Cell Res Ther. 2022 Aug 5;13:403. doi: 10.1186/s13287-022-03042-4 (PMC9356430; doi:10.1186/s13287-022-03042-4)
Supplement: Supplementary file 1 — Additional file 1. Supplemental material 1. [file 13287_2022_3042_MOESM1_ESM.docx]

Follistatin-like 1 guarantees the immunomodulatory properties of mesenchymal stem cells during liver fibrotic therapy

Xiaohong Zheng^1, 2^***, Xia Zhou^1^***, Gang Ma^1^***, Jiahao Yu^1^, Miao Zhang^1^, Chunmei Yang^1^, Yinan Hu^1^, Shuoyi Ma^1^, Zheyi Han^1^, Wen Ning^3^, Boquan Jin^2^, Xinmin Zhou^1^*^#^*, Jingbo Wang^1^*^#^*, Ying Han^1^*^#^*

1. Xijing Hospital of Digestive Diseases, State Key Laboratory of Cancer Biology, Fourth Military Medical University, Xi’an 710032, China
2. Department of Immunology, Fourth Military Medical University, Xi'an 710032, China.
3. State Key Laboratory of Medicinal Chemical Biology, College of Life Sciences, Nankai University, Tianjin, 300071, China
4. **, these authors contributed equally;*
5. *^#^ Correspondence*: Ying Han: [hanying1@fmmu.edu.cn](mailto:hanying1@fmmu.edu.cn)
6. Xinmin Zhou: zhouxmm@fmmu.edu.cn
7. Jingbo Wang: jimberw@163.com
8. Ying Han, MD, Professor
9. Xijing Hospital of Digestive Diseases, State Key Laboratory of Cancer Biology, Xijing Hospital, Fourth Military Medical University, 127 Changle West Road, Xi’an 710032, P.R.China***,*** Tel: +86-29-84771509***,*** Fax: +86-29-82539041***,***
10. Email: [hanying1@fmmu.edu.cn](mailto:hanying1@fmmu.edu.cn)
11. **Keywords:** FSTL1, mesenchymal stem cells, liver cirrhosis, cell therapy, immunosuppressive

**Supplementary**

**Figure S1**


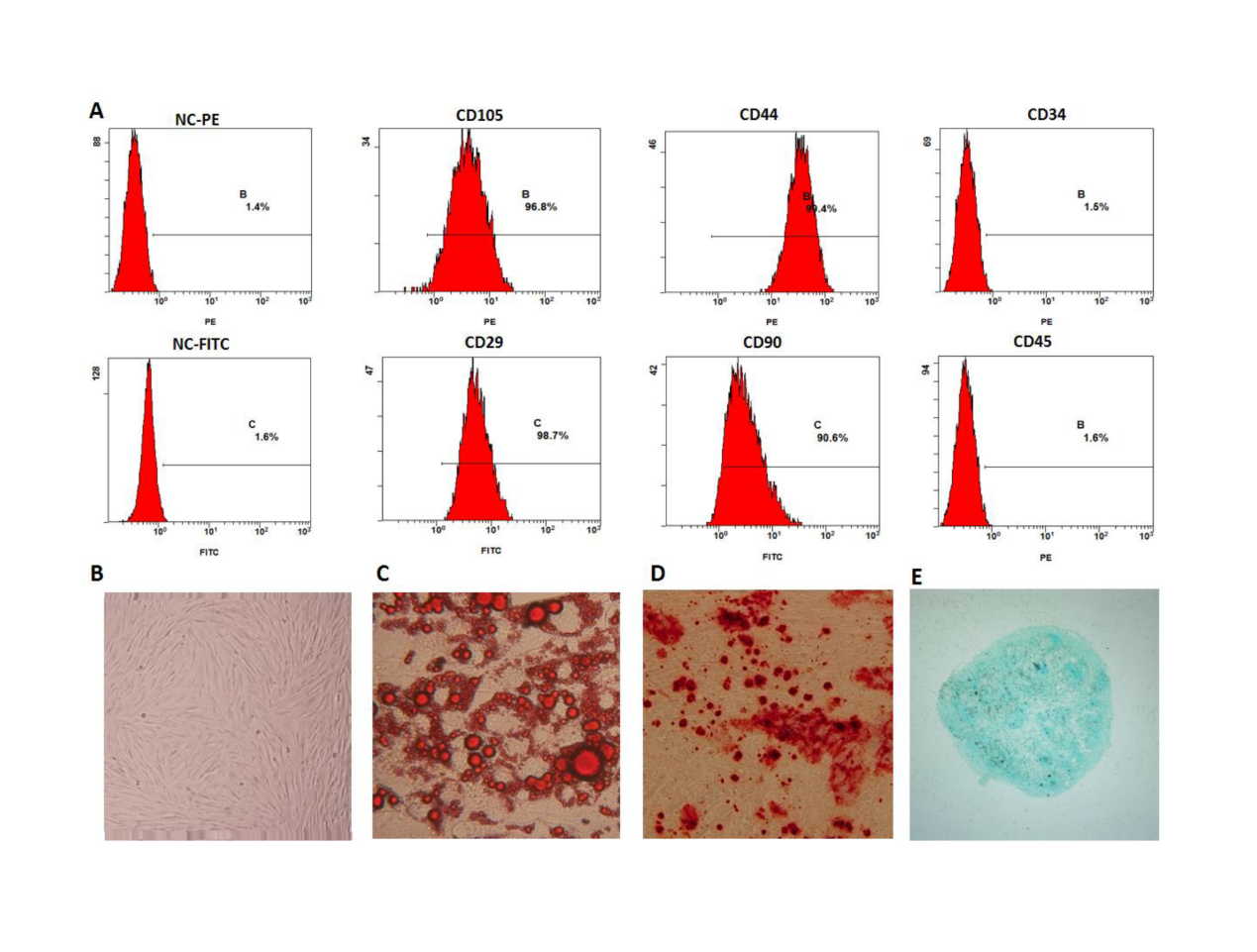


**Fig. S1. Human UC-MSC identification** (A) Human UC-MSC identification.B-E: Assessment of human UC-MSC (B) morphology, (C) osteogenic, (D) adipogenic and (E)chondrogenic abilities.

**Figure S2**


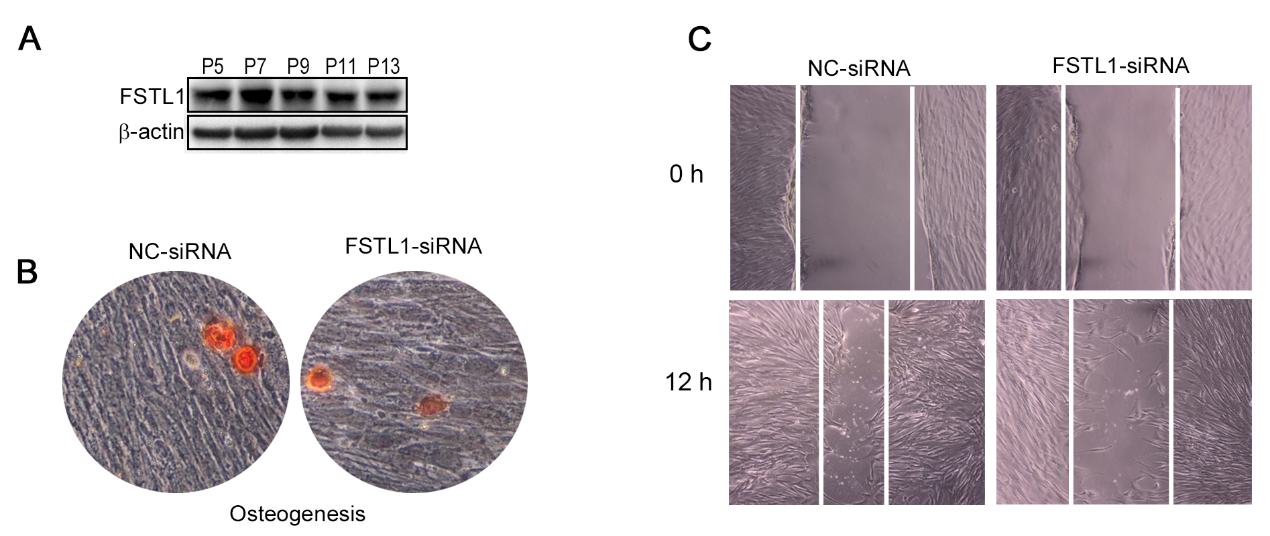


**Fig. S2. Evaluation of osteogenesis and migration after FSTL1 knockdown. (A)** FSTL1 expression; **(B)** osteogenesis; **(C)** Would healing.

**Figure S3**


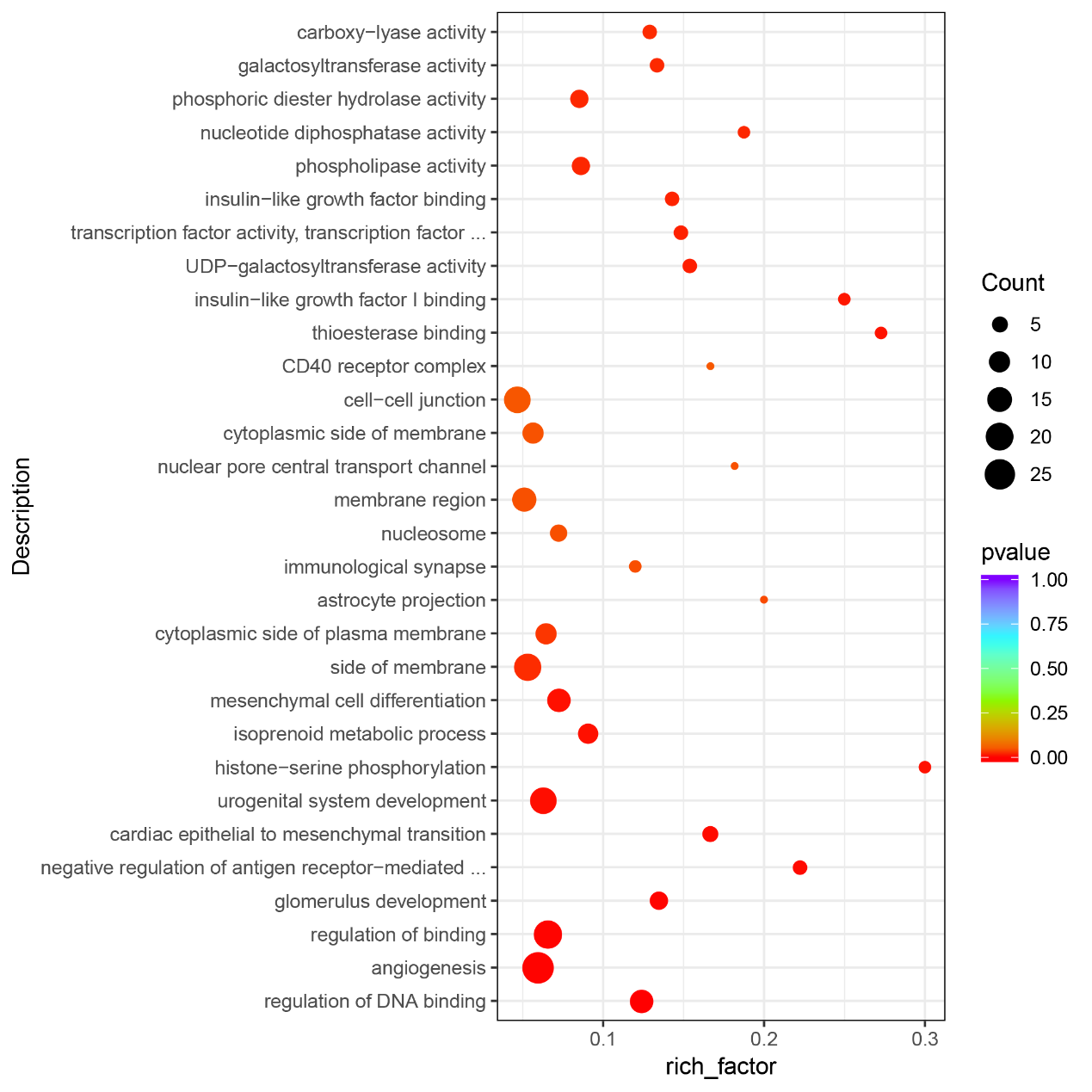


**Fig. S3.** Gene ontology (GO) analysis of the DEGs upregulated in Fstl1^low^ MSCs

**Figure S4**


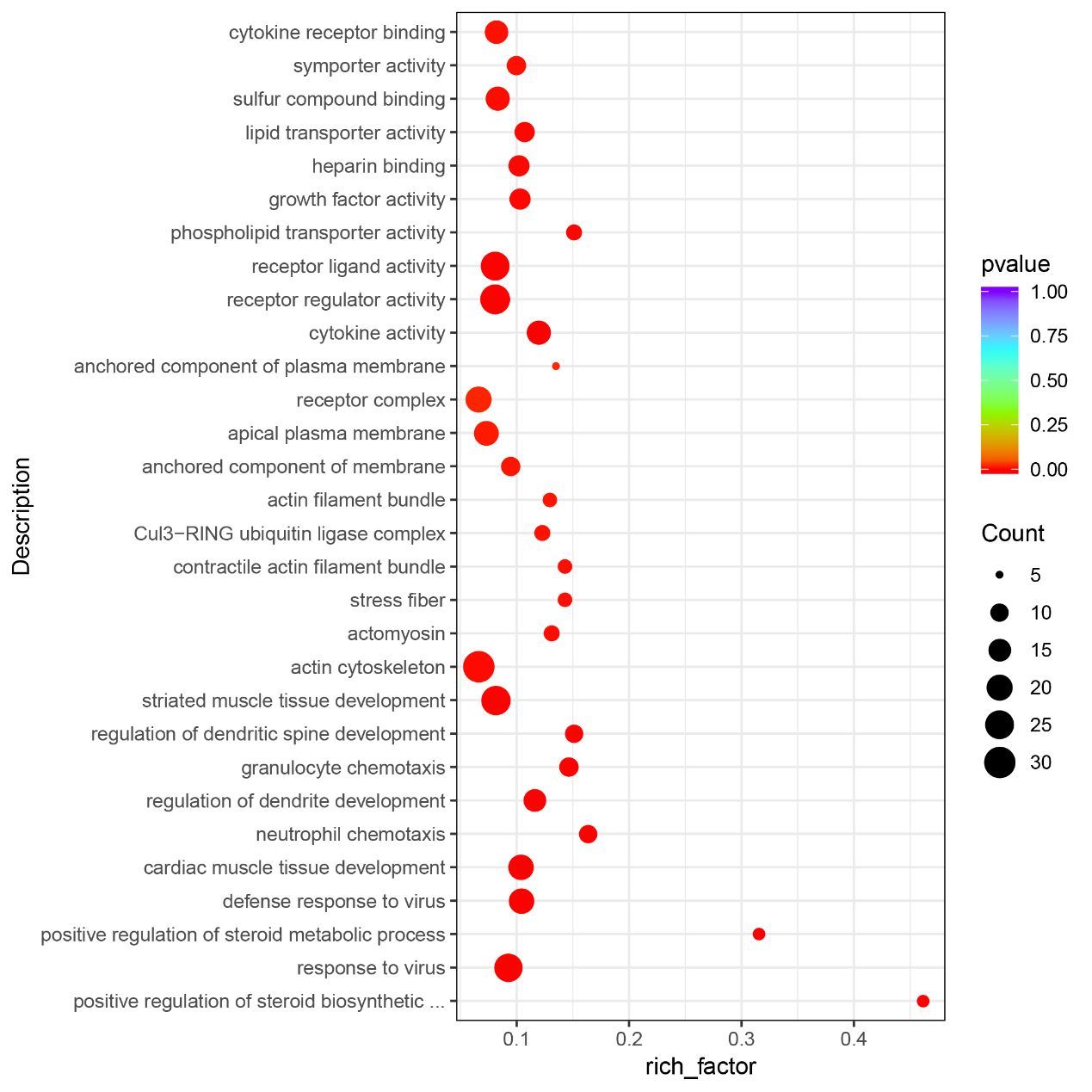


**Fig. S4.** GO pathway enrichment analysis of the DEGs downregulated in Fstl1^low^ MSCs.

**Table 1 Primer sequence**

| **Gene Names** | **Sequence** |
| --- | --- |
| human FSTL1-F | TCTGTGCCAATGTGTTTTGTGG |
| humanFSTL1-R | TGAGGTAGGTCTTGCCATTACTG |
| mouse b-actin F | CATCCGTAAAGACCTCTATGCCAAC |
| mouse b-actin R | ATGGAGCCACCGATCCACA |
| mouse iNOS-F | GCAGAGATTGGAGGCCTTGTG |
| mouse iNOS-R | GGGTTGTTGCTGAACTTCCAGTC |
| mouse TNFa-F | CAGGAGGGAGAACAGAAACTCCA |
| mouse TNFa-R | CCTGGTTGGCTGCTTGCTT |
| mouse Arg-1F | AGACAGCAGAGGAGGTGAAGAG |
| mouse Arg-1R | CGAAGCAAGCCAAGGTTAAAGC |
| mouse IL-10 F | CCCTTTGCTATGGTGTCCTT |
| mouse IL-10 R | TGGTTTCTCTTCCCAAGACC |
| mouse Mrc-1 F | AAACACAGACTGACCCTTCCC |
| mouse Mrc-1 R | GTTAGTGTACCGCACCCTCC |
| mouse Col1a1 F | CCAAGAAGACATCCCTGAAGTCA |
| mouse Col1a1 R | TGCACGTCATCGCACACA |
| mouse a-SMA F | GCTGGTGATGATGCTCCCA |
| mouse a-SMA R | GCCCATTCCAACCATTACTCC |
| mouse TGF-b1 F | TTGCTTCAGCTCCACAGAGA |
| mouse TGF-b1 R | GTTGGACAACTGCTCCACCT |
| mouse Col3a1-F | GACCAAAAGGTGATGCTGGACAG |
| mouse Col3a1-R | CAAGACCTCGTGCTCCAGTTAG |
| mouse IL6-F | TACCACTTCACAAGTCGGAGGC |
| mouse IL6-R | CTGCAAGTGCATCATCGTTGTTC |
| human IDO F | GCCCTTCAAGTGTTTCACCAA |
| human IDO R | CCAGCCAGACAAATATATGCGA |
